# Talin variant P229S compromises integrin activation and associates with multifaceted clinical symptoms

**DOI:** 10.1093/hmg/ddac163

**Published:** 2022-07-21

**Authors:** Latifeh Azizi, Lorena Varela, Paula Turkki, Vasyl V Mykuliak, Sanna Korpela, Teemu O Ihalainen, Joseph Church, Vesa P Hytönen, Benjamin T Goult

**Affiliations:** Faculty of Medicine and Health Technology, Tampere University, Tampere, Finland; School of Biosciences, University of Kent, Canterbury CT2 7NJ, UK; Faculty of Medicine and Health Technology, Tampere University, Tampere, Finland; Fimlab Laboratories, Tampere, Finland; Faculty of Medicine and Health Technology, Tampere University, Tampere, Finland; Faculty of Medicine and Health Technology, Tampere University, Tampere, Finland; Faculty of Medicine and Health Technology, Tampere University, Tampere, Finland; Clinical Immunology and Allergy, Children’s Hospital Los Angeles, Los Angeles, CA, USA; Faculty of Medicine and Health Technology, Tampere University, Tampere, Finland; Fimlab Laboratories, Tampere, Finland; School of Biosciences, University of Kent, Canterbury CT2 7NJ, UK

## Abstract

Adhesion of cells to the extracellular matrix (ECM) must be exquisitely coordinated to enable development and tissue homeostasis. Cell–ECM interactions are regulated by multiple signalling pathways that coordinate the activation state of the integrin family of ECM receptors. The protein talin is pivotal in this process, and talin’s simultaneous interactions with the cytoplasmic tails of the integrins and the plasma membrane are essential to enable robust, dynamic control of integrin activation and cell–ECM adhesion. Here, we report the identification of a *de novo* heterozygous c.685C>T (p.Pro229Ser) variant in the *TLN1* gene from a patient with a complex phenotype. The mutation is located in the talin head region at the interface between the F2 and F3 domains. The characterization of this novel p.P229S talin variant reveals the disruption of adhesion dynamics that result from disturbance of the F2–F3 domain interface in the talin head. Using biophysical, computational and cell biological techniques, we find that the variant perturbs the synergy between the integrin-binding F3 and the membrane-binding F2 domains, compromising integrin activation, adhesion and cell migration. Whilst this remains a variant of uncertain significance, it is probable that the dysregulation of adhesion dynamics we observe in cells contributes to the multifaceted clinical symptoms of the patient and may provide insight into the multitude of cellular processes dependent on talin-mediated adhesion dynamics.

## Introduction

Adhesion of cells to the extracellular matrix (ECM) is a critical part of the development of multicellular organisms and is tightly regulated by multiple signalling pathways. Integrins are the primary ECM receptors and in response to the correct signals support the adhesion of cells to the ECM, maintaining tissue integrity. Integrins are ${\alpha\beta}$-heterodimers and comprise a large extracellular domain that engages the ECM. Each subunit contains a single transmembrane helix and a short cytoplasmic tail that couples the integrin-ECM complexes to the cytoskeleton and signalling machinery. Integrins can exist in low or high affinity states for the ECM, and integrin activation is needed for the maturation of adhesion complexes. The process is exquisitely regulated to ensure correct functioning and to allow the modulation of adhesion strength, critical for processes that require dynamic assembly and disassembly of these attachments (1,2). Tight regulation of integrin activation is important in the regulation of cell cycle, cell migration and survival affecting vital processes such as differentiation, development and immunity.

**Figure 1:**
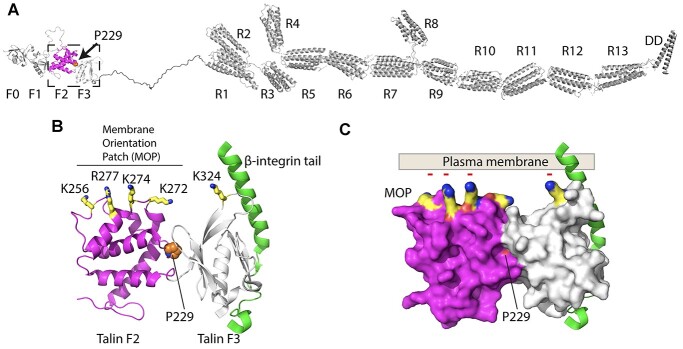
Talin–integrin interaction in the vicinity of the plasma membrane. (**A**) Structural model of talin1. Talin has 2541 amino acids organized into 18 domains. The location of proline 229 is highlighted between the F2 (magenta) and F3 (white) domains of the talin head. (**B**) Cartoon of the F2F3 domains bound to the β-integrin cytoplasmic domain (green). The location of proline 229 (orange) in the interface between F2 and F3 is shown. The basic residues in the membrane orientation patch (MOP) on F2 (yellow) are highlighted. (**C**) Surface representation of (B) showing the P229 is buried in the F2-F3 interface. The negatively charged plasma membrane is shown to illustrate the tripartite interaction between talin F2F3, the β-integrin and the plasma membrane that needs to occur to get robust integrin activation.

Talin is a core component of ECM-adhesion complexes and is essential for the activation and regulation of integrin activity (3,4). It is a large ~250 kDa protein consisting of a head domain that is responsible for integrin binding, an unstructured neck region, a large rod region comprised of 13 helical bundles, R1-R13, and a C-terminal dimerization domain (DD) (Fig. 1A) (5). The R13 and the dimerization domain mediate the primary actin binding and allow the tensile force-dependent stretching of talin (6). The talin rod domains R1-R13 serve as mechanical binary switches that interact with numerous proteins dictated by force-mediated conformational changes in the alpha-helical bundles exposing or disrupting different binding sites such as vinculin (7–9), DLC1 (10,11), CDK1 (12), RIAM (10) and KANK proteins (13,14), reviewed in (15).

The need for tightly regulated integrin adhesion is illustrated by the complex signalling that controls this process, both from inside the cell (inside-out signalling) or from outside (outside-in) (reviewed in 1). Integrin activation and clustering require tightly coordinated interactions between the talin head subdomains, the integrin tails and the plasma membrane, and the protein kindlin (16,17). Disruption of the interaction between talin and integrin and/or the plasma membrane prevents controlled integrin signalling and thereby cell adhesion. Therefore, while mutations in individual integrins may generate rather specific phenotypes centred on that specific integrins function (reviewed in 18), a mutation in talin that heavily influences talin-mediated integrin activation would be predicted to be lethal, unless leading to very subtle changes in integrin regulation (19). However, such a talin variant would impact on the function of all integrins that are regulated by talin.

The talin head domain comprises four subdomains, F0, F1, F2 and F3, which are all required for robust integrin activation (20). While F3 contains the phosphotyrosine-binding (PTB) domain that directly binds to the NPxY motif of most β-integrin cytosolic domains, the F0-F2 domains bind with anionic lipids such as phosphatidylinositol 4,5-bisphosphate (PIP2) in the vicinity of the integrin (21). Mutations in the F0 and F1 domains that disrupt the interaction with Rap1 perturb the integrin activation process (22–24). Similarly, the extended loop within the F1 subdomain of talin head also contributes to integrin activation via direct interactions with integrin (25,26) and the membrane (27). In all the talin head structures resolved to date the F0F1 and F2F3 domains are fixed double domain modules where the orientation between the domains is mediated by extensive interactions. The fixed arrangement of the F2F3 domains has been reported to be important for activation as the F2 domain contains a basic surface, the membrane orientation patch (MOP), that binds tightly to the plasma membrane (4). Upon binding to the integrin tail via the F3 domain, the F2F3 module rotates to engage the MOP with the plasma membrane and this rotation applies torque on the integrin tail to stabilize the active conformation (Fig. 1). Mutations in the MOP region prevent integrin activation (4).

We previously studied the effects of cancer-associated point mutations within talin as reported in the COSMIC database and found talin variants that affect cell morphology, cell migration and cell proliferation (28). Despite the large size of talin and the presence of numerous interaction partners, a single point mutation targeting a critical position can have drastic and even crippling effects on talin functionality and cell adhesion. An example of this is seen with point mutations in the dimerization domain that disrupt dimerization and lead to a loss of cell spreading (28). Similarly, a genetic screen in Drosophila identified a mutant G334E (equivalent to G331E in human talin1) that causes severe morphogenetic defects (29). G334E is located at the interface between the F2 and F3 talin head domains, located on the F3 face. Similarly, loss of talin autoinhibition in flies with a single point mutant, E1777A (30) leads to lethal morphogenetic defects.

These findings suggest that whilst mutations appearing somatically that dysregulate adhesion dynamics might be advantageous for cancer cells, germline mutations that disrupt talin function are likely embryonic lethal (talin1 deficiency is lethal in mice (31)). This is supported by the fact that until recently talin has not had any gene-disease associations, although reports of disease-causing mutation in the *TLN1* gene have emerged, which challenge this picture, including a number of cardiovascular disease-associated mutations in the *TLN1* gene; spontaneous coronary artery dissection (SCAD) (32) and thoracic aortic aneurysm (33). Further, a heterozygous *TLN2* mutation, S339L in F3, was also reported as the cause of nonsyndromic Camptodactyly, which also presented with mild cardiac symptoms, including sinus arrhythmia and sinus bradycardia (34).

We recently identified a patient with a very rare and unusual, complex phenotype. Having excluded all established disease-causing genes involvement in the probands complex checklist of symptoms, the *de novo* TLN1 variant presented in this work was then characterized. Here, we report this novel p.P229S variant and link the human genetics to the biophysical and cell biological consequences to demonstrate the disruption of adhesion dynamics that result from disturbance of the F2-F3 domain interface in the talin head.

## Results

### Case summary—identification of a *de novo* heterozygous variant in the *TLN1* gene

A 20-year-old man of Mexican ancestry was first evaluated at six years of age for thrombocytopenia, T lymphopenia, and low IgG levels. He was born at 35 weeks gestational age and a birth weight of 2100 g. He spent his first month in neonatal intensive care for respiratory distress and persistent thrombocytopenia. Although his platelet count remained approximately 20 000/mcL throughout his course, he never had a serious bleeding episode. At approximately 10 years of age to the present, he continued to experience intermittent sinusitis, otitis media and bronchitis. Treatment with oral antibiotics for each of these multiple episodes resulted in clearance of each clinical infection. He never experienced infections characteristic of T-cell or neutrophil dysfunction.

At 18 years of age, he developed intermittent pain that shifted focus regularly. He had abdominal pain at times diagnosed as small intestinal bacterial overgrowth, headaches often treated as migraines, and joint pain with limited signs of active arthritis. Multiple imaging and laboratory studies failed to identify a cause of these complaints, and an empiric trial of hydroxychloroquine for a possible autoimmune process did not alter his symptoms.

At 19 years of age, he complained of keloid-like skin changes at the sites of very mild linear skin abrasions. The family history was notable for hidradenitis suppurativa in his father and sister who were shown to share a pathogenic variant in *NCSTN* with the patient. Additional problems have included bilateral congenital cataracts, intermittent eczema and intermittent haematuria, the latter judged by Haematology consultation as not related to his chronic thrombocytopenia.

Upon multiple physical examinations, the patient typically appeared alert, cooperative and well-oriented, but generally complaining of moderate to severe headache, abdominal or joint pain. His vital signs were unremarkable. There was rarely evidence of active respiratory tract infection. There was no hepatosplenomegaly or lymphadenopathy. His skin examination revealed mild eczema and multiple linear, narrows, keloid-like scars on his arms and feet.

Abnormal laboratory findings (performed at the Children’s Hospital Los Angeles Clinical Laboratory) over the patient’s history have revealed the following:

1. Thrombocytopenia: generally <20 000/mcL but without significant bleeding
2. Lymphopenia: absolute lymphocyte counts <1000/mcL and low absolute T-cells
3. Recent B-cell subset analysis revealed 96% naïve and 4% switched memory B-cells
4. Initial serum immunoglobulin levels at six years of age included: IgG 273, IgA 130, IgM 36.

Because of poor antibody responses to pneumococcal vaccine, he was started on immunoglobulin replacement therapy, which he has continued to the present.

We acknowledge that the case study presented here may appear somewhat confusing, but this is because we have intentionally provided all the symptoms besides the primarily haematological/immunological presentation. We also describe the lines of investigation we have undertaken during our long-standing oversight of the patients’ treatment in our efforts to determine the basis of this disease of unknown cause. The lack of a simple narrative in this section partially reflects the diverse ailments of the patient, and that we have not yet identified a satisfactory explanation to describe the cause of this complex condition or the diverse presentation.

When he was 16 years of age whole exome sequencing was performed at the Children’s Hospital of Los Angeles Centre for Personalized Medicine Clinical Genomics Laboratory, which is certified under the Clinical Laboratory Improvement Amendments as qualified to perform high complexity clinical laboratory testing. It did not reveal a likely genetic aetiology for his immunologic changes. Most recently, re-analysis of his exome revealed a *de novo*, heterozygous variant in *TLN1* (c.685C > T, p.229Pro > Ser) that was not seen in the parents’ exomes thus confirming the *de novo* nature of the variant and leading to *in vitro* assessment of the variant’s function.

### Biophysical characterization of the P229S and P229L mutations on the structure of F2F3

P229 is located within the F2 domain of the talin head at the interface between F2 and F3 (Fig. 1). The F2F3 domains mediate the interaction with the membrane proximal region of the β-integrin cytoplasmic tail and with the plasma membrane (3,4). Our earlier analysis of the COSMIC database (28) identified a P229L mutation at this site that has the potential to be deleterious. To understand the consequences of these mutations, we set out to compare the biochemical characteristics of wildtype, P229S and P229L talin and their biological function in cells.

**Figure 2:**
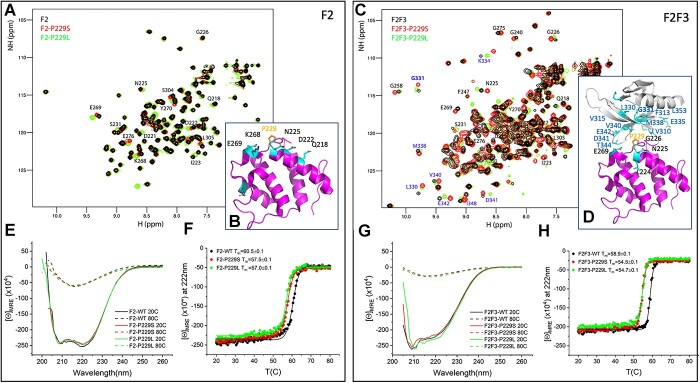
Biochemical characterization of the talin1 F2 and F2F3 domains (black) and the P229S (red) and P229L (green) variants. (**A**, **C**) 2D ^15^N-SOFAST HMQC spectra recorded at 25°C of F2 (A) and the F2F3 double domain (C) showing part of the backbone assignment of the WT. (**B**, **D**) Chemical shift mapping of residues perturbed by the mutations (cyan) on the crystal structure of the F2 domain (B) (magenta) and the F2F3 domains (D) (magenta and grey, respectively). Residue P229 is highlighted in orange. (**E**, **G**) CD far UV spectra at 20 and 80°C of the F2 domain (E) and the F2F3 domain (G) showing no effect of the mutations in the overall secondary structure. (**F**, **H**) Melting curves recorded monitoring the CD signal at 222 nm of the F2 (F) and F2F3 (H) proteins. The calculated melting temperatures (°C) for each protein are shown in the legend. The CD signal is represented as molar ellipticity, [Θ] in deg·dmol^−1^.cm^2^.

To characterize the effect of the P229S and P229L mutations, we generated the following F2 and F2F3 constructs; F2-WT, F2-P229S and F2-P229L, and F2F3-WT, F2F3-P229S and F2F3-P229L. All these recombinant proteins expressed and purified well without signs of proteolysis suggesting that the mutations do not perturb the folding of the domains. This was in stark contrast to the G331E mutation located near to residue 229 where the F2F3-G331E protein was far more proteolytically sensitive (29). We next performed circular dichroism (CD) to determine the secondary structure composition and thermal stability of each protein. Far-UV spectra were collected at 20°C and showed similar alpha helical composition for F2-WT, F2-P229S and F2-P229L (Fig. 2E). Similarly, the F2F3-WT, F2F3-P229S and F2F3-P229L all showed similar secondary structure composition of beta strand and alpha helix (Fig. 2G). These results show that neither the P- > S nor the P- > L mutation cause significant change to the overall secondary structure of the domains. We next measured the thermal stability of each F2 (Fig. 2F) and F2F3 (Fig. 2H) variant by recording the ellipticity at 222 nm from 20 to 80°C. The unfolding profile of all the constructs was similar showing cooperative unfolding, although a small but significant decrease in the melting temperature (Tm) of the mutated proteins was observed, which was more pronounced in the case of the F2F3 domain (P229S, ΔTm = −4.4°C) than in the F2 domain (P229S, ΔTm = −3.0°C). This suggests that the P229 mutations cause a subtle but significant effect on the overall protein stability.

To gain insights into the perturbations caused by each mutation on the tertiary structure of the F2 and F2F3 domains, we carried out NMR collecting ^15^N SOFAST-HMQC spectra of F2-WT, F2-P229S and F2-P229L (Fig. 2A) and F2F3-WT, F2F3-P229S and F2F3-P229L (Fig. 2C). The overall protein folding is very similar in all variants confirming that the folding of both the F2 and the F3 domains is maintained. However, significant shifts in a number of peaks are observed indicative of a local alteration in structure. Using chemical shift mapping, the peaks shifted by the mutation were mapped onto the structures of the F2 and F2F3 domains (taken from pdb 3IVF (35)). Only residues in close proximity to the mutation showed significant chemical shift changes (Fig. 2B, D). In the case of the F2F3 double domain, the mutations in F2 caused significant changes in the proximal residues on both sides of the F2 and F3 interface. This biophysical analysis confirms that both P229S and P229L mutations cause a subtle but specific effect on the structure of the F2F3 double domain affecting residues on either side of the F2F3 interface, and this perturbation leads to a decrease in the thermal stability of F2F3.

### Molecular dynamics simulations suggest that P229S reduces F2F3 interaction affinity

To investigate how mutations affect the F2F3 domain interface, we employed non-equilibrium alchemical MD simulations. The method allows to quantitatively estimate free energy changes in protein thermostability or protein–protein binding upon mutation, using extensive atomistic simulations. The calculations involve relaxation of both WT and mutant forms of a protein, followed by numerous alchemical (non-physical) simulations for morphing of the system from WT to mutant forms and opposite, to sufficiently sample multiple conformational states for reliable free energy estimation (36). The whole procedure was performed for both F2 and F2F3 double domains to predict the change in F2–F3 interaction energy. We did not observe major changes in protein conformation or obvious disturbances in F2–F3 interface. However, mutation P229S reduced the F2F3 interaction energy by 5.6 ± 2.2 kJ/mol, suggesting a modest but consistent destabilization of the interface. Such effect was not detectable in the case of P229L (0.1 ± 1.2 kJ/mol).

### Truncated versions of talin can be used to study adhesion maturation in a simplified setting

**Figure 3:**
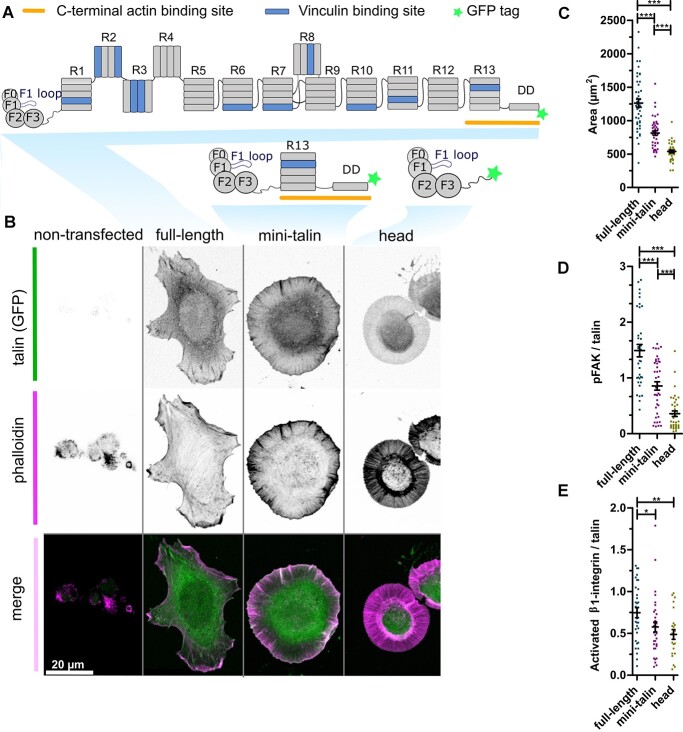
Mini-talin enables cell spreading and integrin-mediated cell signalling. (**A**) Schematic illustration of the full-length talin and the truncated talin proteins, mini-talin and talin head. (**B**) Representative images of TLN1^−/-^TLN2^−/−^ cells expressing full-length talin, mini-talin and talin head. Phalloidin staining is used to visualize the actin cytoskeleton. Non-transfected cell is shown as control. (**C**) Analysis of the cell area for the cells expressing various talin versions, *n* ~ 40 cells pooled from three independent experiments. (**D**, **E**) Fluorescence intensity analysis for adhesion localized pTyr397 FAK (D) and activated β1 integrin relative to talin intensity (E). *n* ~ 35 cells pooled from three independent experiments. The statistical analysis in (C), (D) and (E) was done by *t*-test Mann–Whitney test; ^*^P < 0.05, ^*^^*^P < 0.01, ^*^^*^^*^P < 0.001. Data represent the mean values with SEM.

To understand the phenotypic effects of the talin1 variants at the cellular level, we used talin1 and talin2 knock-out cells (16). Talin knockout cells are unable to adhere on substrate, show low levels of FAK phosphorylation and are unable to activate integrins (16), but these defects can be rescued by transfection with full-length WT talin. In order to study different aspects of talin function in these cells, we also used three previously characterized versions of talin: full-length talin, talin head and mini-talin (Fig. 3A) (37). To learn about talin-integrin interface, we used the talin head construct, which contains the integrin-binding site in F3, but due to lack of the C-terminal rod region, it cannot form the link to the actin cytoskeleton (Fig. 3A and B). To get information about the early talin-mediated integrin activation events, we used mini-talin (Head-R13-DD), which contains the integrin-binding site in F3 connected to the actin binding region R13-DD. Therefore, mini-talin can facilitate force transmission between the cell cytoskeleton and the integrin-ECM complexes, leading to integrin activation. However, mini-talin lacks the talin rod domains R1-R12 and the diverse signalling molecules that they recruit as a function of mechanical load, leading to a simplified model for studying talin head–integrin interaction in detail.

Talin knockout cells re-transfected with full-length WT talin polarize and spread to an average area of 1250 μm^2^ (Fig. 3C). Full-length expressing cells also showed high auto-phosphorylation of FAK and clustering of activated integrin within the talin-rich adhesions (Fig. 3D and E). Mini-talin expressing cells showed isotropic spreading with average area of 750 μm^2^ (Fig. 3C), decreased FAK phosphorylation and integrin activation (Fig. 3D and E), as expected when the rod bundles and their interactions that allow the full maturation of focal complexes are omitted. The talin head alone lacked the C-terminal actin binding site and therefore lacks the force-transmission ability of talin, and as a result, the cell area decreased to an average of 500 μm^2^ and FAK signalling and integrin activity were almost lost (Fig. 3D and E).

### P229S variant causes significant defects in cell migration

Having characterized the three talin constructs, we next introduced the P229S and P229L point mutations into each of them. Western blot analysis confirmed that the cells produced similar amount of each of these proteins at the correct molecular weights (Supplementary Material, Fig. S1A).

**Figure 4:**
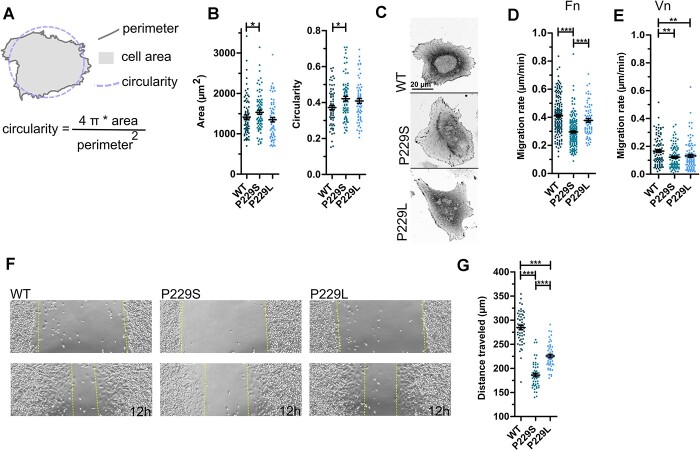
Talin variants P229S and P229L compromise cell spreading, migration and wound closure. (**A**) Schematic illustration of the morphological parameters of a cell; the area covered by the cell is known as cell ‘area’; the distance around the cell is called ‘perimeter’ and the normalized ratio of cell area and the perimeter is called ‘circularity’. (**B**) Cell area and circularity measured for the cells expressing full-length versions of talin WT, P229S and P229L. *n* ~ 70 cells pooled from three independent experiments for each set of analysis. (**C**) Representative images of cells expressing full-length talin proteins (WT, P229S, P229L). (**D**) Random migration analysis of the cells transfected with full-length talin WT, P229S and P229L on fibronectin (10 μg/ml) (Fn) coated surface. *n* ~ 95 cells pooled from three independent experiments. Experiment performed in 10% FBS medium. (**E**) Random migration analysis of the cells transfected with full-length talin WT, P229S and P229L on vitronectin (10 μg/ml) (Vn) coated surface. *n* ~ 90 cells pooled from three independent experiments. Experiment performed in 10% FBS medium. (**F**) Representative images of the wound closure assay for the cells transfected with full-length talin WT, P229S and P229L in 0 h (upper) and after 12 h (lower). (**G**) The distance travelled (μm) by the cells to close the wound, was calculated by analyzing the scratched area from 0 h and after 12 h. *n* ~ 25 scratches from three independent experiments. The statistical analysis in (B), (D), (E) and (G) was done using *t*-test Mann–Whitney test; ^*^P < 0.05, ^*^^*^P < 0.01, ^*^^*^^*^P < 0.001. Data represent the mean values with SEM.

Talin-knockout cells transfected with full-length P229S have a slightly larger surface contact area (*P*-value 0.045) and have higher circularity (*P*-value 0.019) in comparison with cells transfected with full-length WT (Fig. 4A–C). P229L caused less severe changes to these characteristics (Fig. 4A–C). Next, the influence of P229S on the cells ability to polarize and accommodate a restricted footprint was quantified using a single-cell approach, where cells are grown on micro-engineered cell adhesive islands with an ‘ice-cream’ shape of 400 μm^2^ surface area (Supplementary Material, Fig. S2A). This analysis did not show statistically significant differences between WT and P229S, but a slight decrease in cells ability to cover the pattern was noticed with P229S-bearing cells (Supplementary Material, Fig. S2A).

Analysis of the random migration of individual cells on either fibronectin (Fig. 4D) or vitronectin (Fig. 4E) revealed that cells expressing the P229S variant showed a significant decrease in migration rate compared to WT in full serum media. In addition, the cell migration speed of both P229S and P229L transfected cells on vitronectin in serum-free medium was decreased in comparison with WT (Supplementary Material, Fig. S2B). Interestingly, P229L did not significantly affect the cell migration rate on fibronectin when compared to WT (Fig. 4D). Next, to understand the effect on collected cell migration, we studied the cells with a wound closure assay. The delay in the closure of the scratch with both mutants was already evident within 12 h. With P229S, the effect was more pronounced as, after 12 h, the cells had travelled only three-quarters of the distance compared to the WT (Fig. 4F and G). After 20 h, WT and P229S transfected cells, had filled ~96 and ~61%, respectively, of the scratch length. Altogether, these results showed that the P229S variant has a clear negative effect on the cell migratory capacity.

### P229S leads to disrupted adhesion signalling

Integrin-mediated adhesions start as nascent adhesions at the cell leading edge, where several crucial signalling molecules, including FAK and paxillin, are recruited to the adhesions and subsequently phosphorylated (38). Some nascent adhesions are short-lived (39) but with correct signals some grow and mature into larger focal complexes. As the adhesion mature, and more paxillin and FAK get recruited and phosphorylated, monitoring phosphorylation of paxillin and FAK allows for quantifying adhesion maturation. Similarly, the maturation and turnover rate of the adhesions lead to changes in cell size, polarization and migration ability that can also be measured as read-outs of these signalling events. Therefore, to understand the defects in cell migration and adhesion signalling caused by the variant P229S, we next assessed the maturation of the adhesion complex by measuring; (i) the auto-phosphorylation status of FAK, (ii) the degree of integrin activation within the adhesions, (iii) paxillin localization to adhesions (iv) phosphorylation status of paxillin at tyrosine 31 (pY31) and (v) the average size of adhesion complexes at the cell boundary, all established readouts of adhesion signalling.

**Figure 5:**
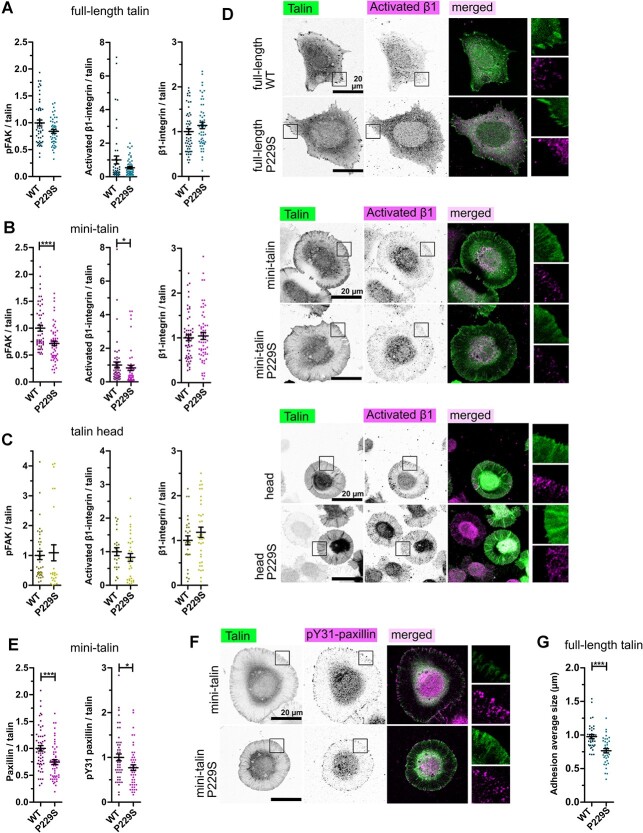
P229S influences integrin activation and adhesion signalling. (**A**) pTyr397 FAK/talin, activated β1-integrin/talin and total β1-integrin/talin intensity ratios for the cells transfected with full-length talin WT and P229S. *n* ~ 50 cells pooled from three independent experiments. (**B**) pTyr397 FAK/talin, activated β1-integrin/talin and total β1-integrin/talin intensities ratios for the cells transfected with mini-talin, WT and P229S. *n* ~ 50 cells pooled from three independent experiments. (**C**) pTyr397 FAK/talin, activated β1-integrin/talin and total β1-integrin/talin intensities ratios for the cells transfected with talin head, WT and P229S. *n* ~ 30 cells pooled from three independent experiments. (**D**) Representative images of cells expressing full-length/truncated talin proteins. Activated β1-integrin is shown in the staining. (**E**) Paxillin/talin and pY31 paxillin/talin intensities of mini-talin data. *n* ~ 50 cells pooled from three independent experiments. (**F**) Representative images of pY31 paxillin/talin intensity of cells expressing mini-talin. (**G**) Adhesion average size quantification (μm) from the periphery area of the cells transfected with full-length talin WT and P229S. The data in (A), (B), (C), (E) and (G) are normalized to the WT in each set. The statistical analysis in (A), (B), (C), (E) and (G) was done by *t*-test Mann–Whitney test; ^*^P < 0.05, ^*^^*^P < 0.01, ^*^^*^^*^P < 0.001. Data represent the mean values with SEM.

In full-length versions of talin WT and P229S variant, we could not see any differences in either pFAK or β1-integrin status within the adhesions (Fig. 5A and D; Supplementary Material, Fig. S1C). Furthermore, no significant difference in the level of vinculin within the adhesion sites was observed between the full-length talin WT and P229S (Supplementary Material, Fig. S1D). However, the mini-talin P229S variant led to a decrease in both pFAK and activated β1-integrin levels within adhesions (Fig. 5B; Supplementary Material, Fig. S1C), suggesting that the complexity of the rod-mediated interactions and signalling might mask the effects of the mutation in the full-length version in cultured cells. With the cells expressing talin head constructs, we could not observe any effects of the mutations, most likely because the overall integrin activation level was already extremely low due to the lack of force-transmitting connection to the actin cytoskeleton via R13-DD (Fig. 5C and D). We next assessed the localization and phosphorylation status of paxillin at tyrosine 31 (pY31) in adhesion sites. We did not see any effect on the total levels of paxillin or phosphorylated paxillin in cells transfected with the full-length constructs (Supplementary Material, Fig. S1E) but in the case of mini-talin, we saw a significant decrease in both paxillin and phospho-paxillin levels when comparing cells expressing P229S to WT (Fig. 5E and F). Together, these results highlight the subtle but consistent disturbance on adhesion signalling as a result of the P229S mutation.

Adhesion signalling further regulates the adhesion maturation process that goes hand in hand with the growth of the adhesion complex (38). To define the influence of mutations on adhesion maturation, we quantified the adhesion number and size around the cell periphery using the paxillin staining from cells expressing the full-length talin constructs. There was a significant decrease in the average size of adhesions in cells expressing P229S variant (P229S: 0.77 μm, WT: 0.98 μm; *P* < 0.0001; Fig. 5G) while the total number of adhesions per cell was slightly higher in the P229S transfected cells in comparison with the WT transfected cells (P229S: 76, WT: 64; *P* = 0.13; Supplementary Material, Fig. S2C). The total adhesion area remained the same for both constructs (Supplementary Material, Fig. S2C) suggesting that although the P229S-expressing cells’ adhesions might be more numerous, they do not reach the size observed in the WT-transfected cells. These results show that the disrupted signalling leads to decrease in adhesion size possibly indicating interference in the adhesion maturation process.

## Discussion

Integrin activation and its connection to actin cytoskeleton is vital for cell-ECM-dependent physiological functions during development, tissue morphogenesis, tissue repair, cancer and immune response (40,41). To initiate the communication between cell interior and ECM, talin is recruited to the plasma membrane, and the head region of talin engaging the integrin leads to integrin activation. Specifically, this interaction is due to the F3 domain binding to the β-integrin cytoplasmic domain, and positively charged residues in F2 and F3 domains, which engage the plasma membrane and directly contribute to integrin activation (4,35,42,43). Here, we report the characterization of a novel talin variant, P229S, which was identified in the tri-exome sequencing of a patient with a complex medical condition. We showed that proline 229 is critical to the intimate juxtaposition of F2F3, and modification to serine (or leucine) disrupts this interface severing the tight linkage between the two domains, resulting in a subtle but specific decrease in protein stability, which manifests as defects in cell movement, integrin activation and adhesion signalling.

### Coordination of talin-integrin-membrane interactions is critical for robust adhesion signalling

**Figure 6:**
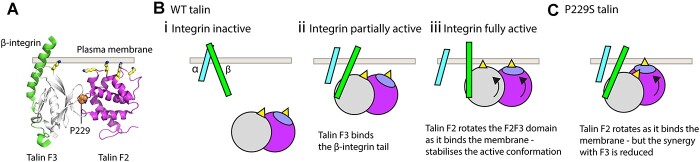
Proposed model for P229 function in the synergistic coordination of the talin-integrin-plasma membrane interactions. (**A**) Cartoon representation of the F2 (magenta) and F3 (grey) bound to the β-integrin tail (green). Proline 229 is shown in orange at the interface between the two domains. The talin F2 and F3 domains work together to induce integrin activation by binding to the β-integrin tail and the plasma membrane simultaneously, the interaction with the membrane orientation patch (MOP; yellow) on F2 is essential for robust integrin activation. (**B**) Model of the integrin activation process mediated via the F2F3 domains (based on the model proposed by (4)). (i) Integrins exist in an inactive state in the absence of talin binding. (ii, iii) The F2F3 double domain module inextricably links integrin binding to membrane binding. To simultaneously optimize the interactions of both F3-integrin and F2-membrane, the F2F3 module needs to undergo a 20° rotation and this torque (iii) is thought to help lock integrin in the active conformation. (**C**) The P229S mutation disrupts the tight apposition of F2 and F3 and uncouples the tight interplay between integrin and membrane binding. With the two domains able to move independently, the optimization of the F2-membrane interface no longer exerts torque onto the F3–integrin interaction perturbing the tight regulation of the talin-mediated integrin dynamics.

The F3 engages the integrin cytoplasmic tail (44), but the F3 domain alone is unable to sustain integrin activation and the entire talin head region, spanning F0-F3, is required for maximal activation (20,26). However, the F2F3 domains alone can partially activate integrins, and the role of a cluster of residues on the F2 domain, comprising K256, K272, K274 and R277, dubbed the membrane orientation patch (MOP) are required to bind to the plasma membrane to sustain robust integrin activation (Fig. 6).

Analysis of the crystal structure of talin bound to integrin revealed that to optimally engage the integrin tail and the plasma membrane simultaneously, a 20° rotation of the F2F3-integrin complex was required (4) leading to a model of integrin activation mediated by the membrane-based reorientation of the integrin tail as a result of F2F3 binding (Fig. 6B). This model (4,45) implicates the role of plasma membrane in the activation process and proposes that the torque generated on the beta tail as the talin head rotates to fully engage the membrane helps to activate the integrin, but also to stabilize the active conformation. This model requires F2F3 to function as a single module such that the rotation of F2 leads to rotation of F3. We hypothesize that P229S mutation reported here perturbs the coupling between F2 and F3 domains, with the consequence that the rotation of F2 to engage the membrane no longer applies enough torque on the F3-integrin to facilitate activation (Fig. 6C). It is the decoupling of membrane binding and integrin binding that we believe is the basis for why the P229S variant disrupts the regulation of integrin activation.

### Mini-talin: a potential tool to detect minor changes in talin head functions

The talin head by itself can bind to and partially activate integrins but talin knockout cells rescued with the talin head alone do not spread well and it is not possible to visualize the adhesive structures that form (Fig. 3A). Measuring integrin activation metrics alone here would not provide the sensitivity required to detect nuanced changes in this process. On the other hand, full-length talin is rich in interactions and contributes to numerous cellular signalling pathways, and therefore modest effects on adhesion dynamics or integrin activation status can be difficult to detect in the context of such complex protein assemblies. This is exemplified in the current study, where, despite the full-length talin P229S construct showing a clear decrease in adhesion size indicative of defects in adhesion maturation and cell migration capacity, it was challenging to pin-point the exact signalling events that were affected. In contrast, the mini-talin P229S construct displayed stalled adhesion dynamics when compared to the wild type, enabling us to visualize the impact of P229S on the adhesion maturation process. We therefore propose that mini-talin represents a suitable molecular tool for the purpose of studying talin variants that impact integrin activation and the dynamic nascent adhesion maturation process. This engineered talin protein couples the talin head to the C-terminal actin binding and dimerization domain in R13-DD (Fig. 3A) and therefore connects the integrin-talin head complexes to the actin cytoskeleton. This construct omits the 12 talin rod domains, R1-R12, whose switch-like behaviours, contribute to the regulation of adhesion strength and signalling (15). In contrast with the head alone, which enables some cell attachment without proper integrin activation, mini-talin enables extensive isotropic cell spreading and leads to integrin activation and nascent adhesion formation, albeit to a lesser extent compared to full-length talin. Therefore, this construct supports the mechanocoupling to actomyosin and enables adhesive structures to be observed meaning that the talin head functions can be explored with a greater precision than using the head alone. This construct (submitted to Addgene) will facilitate the study of talin head interactions in a simplified system in a more quantitative manner than currently available integrin activation assays. It should also enable a more detailed examination of the integrin activation process than the current approaches, and with less multifactorial contributions from the mechanical signalling of the talin rod.

### A subtle talin disruption would manifest in a wide spectrum of conditions

There are many pathogenic integrin variants known to cause specific conditions in processes dependent on the affected integrin. For instance, leukocyte adhesion deficiency (LAD) is caused by a range of mutations in the β2 integrin, and Glanzmann’s thrombasthenia, is caused by numerous mutations in αIIb or β3 integrin (18). However, because talin is an activator and adapter protein for multiple integrins (46), it is expected that a talin variant causing a defect in integrin activation would impact all integrins and so, likely be embryonically lethal. However, the loss of synergy between the F2F3 domains as a result of the P229S mutation is subtle, resulting in a small shift in the biophysical properties of the protein, which manifest as a minor disruption of cellular processes. However, at the organismal level, because the P229S variant maintains the basic talin functions, but subtly influences the fine-tuned balance of integrin activation, we anticipate it will impact on all integrin processes that are talin-dependent. Our data show that the P229S mutation impacts on both β1 and β3 integrin-dependent processes, which suggests that other integrins will be affected as well. Many of the symptoms experienced by the proband involve processes that require talin-dependent integrins. For example, thrombocytopenia has been found to be associated with integrin β3 mutations (47,48) and β3 integrins play a critical role in platelet aggregation (49). Furthermore, integrins are essential to maintain normal myeloid and B lymphoid differentiation (50) and β2-integrins are essential for leukocyte trafficking, immune suppression and immune deficiency disease (51), which could explain the multiple immune system-related defects. Further work would be needed to establish a causal link between the variant identified here and the phenotype of the patient.

It is important to stress that our biophysical, biochemical and cell biology experiments show that the P229S variant affects the function of the talin protein in cells, but we do not have evidence yet that the P229S talin variant is the cause of the multitude of delocalized symptoms affecting the patient. Therefore, we would like to emphasize that this talin variant P229S should be considered a variant of uncertain significance (VUS) until additional cases with similar variants and phenotypes are identified.

In conclusion, we show that talin variant P229S results in subtle perturbation of the talin head structure, that influences the fine control of integrin dynamics leading to disturbance in adhesion maturation and compromised cell migration. As talin coordinates many integrin subtypes, we propose that this modest dysregulation would likely impact many cellular processes leading to numerous symptoms. This study emphasizes the central role of talin as integrin activator and as a link between cytoskeleton and extracellular environment.

## Materials and Methods

### Protein expression and purification

Talin1 F2 and F2F3 constructs in their WT version and with sequence variations P229S and P229L were synthesized into a pET151 plasmid (GeneArt). All constructs were expressed in BL21(DE3) *E. coli* cells and grown at 37°C in LB media or 2 M9 minimal media supplemented with ^15^N-labelled ammonium chloride for NMR. Expression was induced with 1 mm IPTG when OD_600_ reached 0.7 and cells were then incubated at 20°C overnight. Following harvesting, the cell pellets were resuspended in 50 mm imidazole, 500 mm NaCl, and 20 mm Tris–HCl, pH 8 and lyzed using sonication. The proteins were purified by nickel affinity chromatography using a HisTrap HP column (Cytiva) and the his-tag cleaved using TEV protease. The cleaved His-tag and TEV protease were removed using a HiTrap SP HP cation exchange column (Cytiva) (52). All bacterial expression constructs have been deposited in Addgene at http://www.addgene.org/ben_goult.

### CD experiments

Protein samples at a concentration of 50 μm were placed in a quartz cuvette and far-UV spectra were collected between 260 and 200 nm wavelengths using a JASCO J-715 spectropolarimeter. Protein thermal unfolding was monitored by following the change in CD signal at 222 nm between 20 and 80°C.

### NMR characterization

^15^N-labelled protein samples at 0.1–0.3 mm were placed in a Shigemi NMR tube containing 10% D_2_O and 2D ^1^H-^15^N SOFAST-HMQC spectra were collected at 298 K with 256 increments on a Bruker Avance3 spectrometer operating at a ^1^H frequency of 600 MHz equipped with a TCI-P CryoProbe. All spectra were processed using NMRPipe (53) and Bruker Topspin and analysis of the amide backbone chemical shifts and chemical shift mapping was carried out using CcpNmr Analysis (54) and NMRView (55). For the chemical shift mapping, the previously determined backbone assignments (BMRB accession numbers 16 932 (F2F3) and 16 930 (F2) (56)) were used to identify the shifted peaks, which were mapped onto the F2 and F2F3 crystal structures (taken from PDB ID 3IVF (35)) using PyMOL (Schrödinger LLC).

### MD simulations

Alchemical free energy calculations were prepared using PMX software (57) and performed with Gromacs (58) at Mahti supercomputer, CSC, Finland. The Amber99SB^*^–ILDN force field (59) and TIP3P water model in 0.15 M NaCl solution were used. Each system was energy minimized for 10 000 steps and then equilibrated for 1 ns using harmonic position restraints on all heavy atoms of the protein. The temperature and pressure of the system was maintained at 298 K and 1 bar using Berendsen algorithm (60) for the system equilibration, while V-rescale (61) and Parrinello–Rahman (62) algorithms were used for equilibrium MD and non-equilibrium morphing simulations. Integration time step of 2 fs was used in all the simulations. Each state of the system was run for 200 ns equilibrium MD. 186 non-equilibrium morphing simulations were prepared for each physical state of the system, using snapshots captured from the equilibrium trajectories, linearly spaced from 15 to 200 ns. Fast non–equilibrium simulations were morphing the system from one state to another in 100 ps. A soft-core potential (63) was used. The whole calculation, including system preparation, was repeated three times and average free energy value was obtained. The calculated free energy changes upon mutation P229S: F2 (−4.4 ± 0.9 kJ/mol), F2F3 (−4.3 ± 1.2 kJ/mol); P229L: F2 (−2.3 ± 0.6 kJ/mol) F2F3 (3.3 ± 2.2 kJ/mol) were used to calculate the influence of the mutation into the F2F3 interface. 3IVF PDB structure (35) was used for MD.

### Cell line, transfection and talin expression constructs

The TLN1^−/−^ TLN2^−/−^ mouse kidney fibroblast (MKF) cell line used were described previously (16). Cells were maintained in a humidified 37°C, 5% CO_2_ incubator. High glucose Dulbecco’s modified Eagle medium (DMEM) supplemented with 10% fetal bovine serum (FBS) was used in all experiments. The cell line was regularly tested for mycoplasma contamination. Talin variants were subcloned into a modified pEGFP-C1 vector backbone (Clontech). Cells were transfected with 6 μg plasmid DNA per 10^6^ cells using Neon transfection system (Thermo Fisher Scientific) using parameters 1400 V, 30 ms, one pulse. The expression constructs for cell culture experiments with the C-terminal EGFP-tag are as follows: full-length wild-type talin1 (1–2541); mini-talin (1–490 + 2296–2541; head-R13-DD domains); head (1–490) and the P229S variant for each construct. These expression constructs are available with maps and sequences in Addgene (https://www.addgene.org/Vesa_Hytonen/).

### Migration rate analysis and western blotting

Transfected cells were incubated overnight in the cell culture incubator, trypsinized and plated on the well-plates coated with 10 μg/ml fibronectin. Cells were allowed to attach for 90 min, after which the medium was changed. The time-lapse images captured with EVOS FL auto microscope (Thermo Fisher Scientific) were analyzed manually (12 h) using ImageJ (Fiji) and MTrackJ plugin (64,65). Cells analyzed from either fibronectin (10 μg/ml) and vitronectin (10 μg/ml) coated surfaces, were cultured in 10% FBS containing medium. In addition, cells were analyzed in 0% FBS medium on surface coated with vitronectin (10 μg/ml).

For western blot, cells were cultured in the same time range as for migration speed analysis (48 h post transfection), after which they were collected, boiled at 100°C for 10 min and run on the gel for wet blotting. Band intensities were analyzed using ImageJ (Fiji).

### Wound closure assay

Transfected cells were incubated in high confluency (90%) in a 24-well plate for 48 h in the cell culture incubator. The wound (approximately 800 μm) was created using a 100 μl pipette tip. After wounding, the wells were washed with the media to remove dead and detached cells. The wound closure was observed using EVOS FL auto microscope. The images were analyzed manually using ImageJ (Fiji) by using a freehand line tool measuring the distance that cells travelled in 12 h from the starting point.

### Immunostaining and confocal imaging

Transfected cells were incubated overnight in the cell culture incubator, trypsinized and plated on coverslips coated with 10 μg/ml human fibronectin and incubated overnight again. Cells were fixed with 4% paraformaldehyde, permeabilized and immunostained using standard protocol. Antibodies are listed in Supplementary Material, Table S1.

### Image analysis: protein expression level quantification from confocal images

Immunostained samples were imaged with Zeiss LSM800 laser scanning confocal microscope, mounted on inverted Zeiss Axio Observer.Z1 (Zeiss, Oberkochen, Germany) using Plan-Apochromat 63×/1.40, WD 0.19 mm oil immersion objective. For the quantification of adhesion-localized protein intensity, 5–10 adhesion sites per cell were selected based on the EGFP-talin channel and each selection was copied to the red fluorescence channel, followed by measurement of intensities from both channels using ImageJ. Background was assessed from the EGFP channel, from areas devoid of fluorescence signal and these areas were again copied to the red fluorescence channel, followed by measurement of the intensities. The background intensities were subtracted from the measured adhesion protein intensities for each channel.

### Image analysis: quantification of adhesion number and size

For the quantification of adhesion number and size (Supplementary Material, Table S1), samples immunostained against paxillin were used. Paxillin is one of the well-accepted markers of focal adhesions. We used the paxillin single channel image with raw data without any pre-processing. All quantification processes were carried out using ImageJ. Quantification was done with ImageJ as previously shown (66). Shortly, we created an 8-bit image and converted it to greyscale binary image. The peripheral area of the cell was selected using free-hand tool in ImageJ. to analyze particles command with the pre-defined parameters (size = 0.2–5 μm^2^, circularity = 0–1) was then executed, which scans the thresholded (binary) image detecting the adhesion sites. It measures the number and size of each adhesion and provides the total area of the adhesion sites.

### Photoresist lift-off assisted patterning of ECM proteins (LOP)

To analyze the behaviour of a single cells transfected with full length WT and P229S and to see the effect of the mutation on a single cell in detail, we used the method of photoresist lift-off assisted patterning of ECM proteins (LOP). Previously, Moeller *et al*. described the detailed protocol for this method (67). Shortly, the coverslips (18 × 18 mm Paul Marienfeld GmbH & Co. KG Lauda-Königshofen Germany) were cleaned using acetone, isopropanol and MQH_2_O, dried and treated with oxygen plasma for 2 min (Vision 320 RIE, Advanced Vacuum). The photoresist S1818 (Microchem, Westborough, Massachusetts, USA) was spin-coated on the coverslips using the standard contact photolithography and 2 μm thick resist layer was photopatterned (5.6–7.5 mW/cm^2^ at 365 nm, OAI instruments). The ice-cream patterns were designed using a chrome mask. The S1818 structure on the coverslip was treated with oxygen plasma for 15 s at 80 W (PICO plasma cleaner; diener) followed by incubating the S1818 structure with 0.1 mg/ml PLL-g-PEG in PBS solution for 30 min at room temperature. The coverslip was then rinsed with MQ and placed in a previously cleaned beaker for the lift-off procedure, protein coating and cell culturing. The lift-off and cell culturing procedures are described in the Supplementary Material.

## Supplementary Material

P229S_supp_220612_ddac163
