# Supplementary material for: Talin variant P229S compromises integrin activation and associates with multifaceted clinical symptoms
Source: Hum Mol Genet. 2022 Jul 21;31(24):4159–72. doi: 10.1093/hmg/ddac163 (PMC9759328; doi:10.1093/hmg/ddac163)
Supplement: P229S_supp_220612_ddac163 [file p229s_supp_220612_ddac163.pdf]

Supplementary information for

Talin variant P229S compromises integrin activation and associates with multifaceted clinical symptoms

Latifeh Azizi[1‡], Lorena Varela[2‡], Paula Turkki[1,3‡], Vasyl V. Mykuliak[1], Sanna Korpela[1], Teemu O. Ihalainen[1], Joseph Church[4]*, Vesa P. Hytönen[1,3]* and Benjamin T. Goult[2]*

[1]Faculty of Medicine and Health Technology, Tampere University, Tampere, Finland.
[2]School of Biosciences, University of Kent, Canterbury, Kent, CT2 7NJ, UK.
[3]Fimlab Laboratories, Tampere, Finland.
[4]Clinical Immunology and Allergy, Children's Hospital Los Angeles, LA, USA

‡ These authors contributed equally to this work.

*Corresponding authors: Benjamin T. Goult: B.T.Goult@kent.ac.uk, Vesa P. Hytönen: vesa.hytonen@tuni.fi, Joe Church: JChurch@chla.usc.edu

Supplementary Materials and Methods

Photoresist lift-off assisted patterning of ECM proteins (LOP)

Lift-off procedure

Five 25 ml beakers were cleaned using 2% Hellmanex by sonication, rinsed with MQ and let to dry. The coverslip was placed in the beaker with a photoresist surface on top. Each beaker (named 1 to 5) contains a mixture of MQ and N-Methyl-2-pyrrolidone (NMP) and the lift-off procedure was performed in order: 1) 1/3 NMP, 2/3 MQ, 20 seconds, no sonication 2) MQ, 10 seconds, no sonication 3) pure NMP, 1 minute, with sonication 4) pure NMP, 5 minutes, with sonication 5) 1/2 NMP, 1/2 MQ 1 minute, with sonication following by rinsing the coverslips with MQ.

Cell culture, staining and imaging of the LOP samples

Before starting the protein coating and cell culture, the coverslips were sterilized using laminar UV. Coverslips were coated with a mixture of 10 µg/ml fibronectin and 5 µg/ml vitronectin in PBS for 45 minutes. MKF cells were transfected, using the Neon transfection method, with full-length talin WT or P229S a day before. Cells were trypsinized, resuspended in a DMEM medium containing 10% FBS followed by centrifugation (150 g, 5 minutes). They were washed two times with a medium without serum and the cell pellet was again resuspended into the medium without serum. The amount of ~30,000 cell/cm^2 was pipetted on top of the coverslips and incubated in the cell culture laminar for 4 hours after which they were fixed by 4% PFA solution for 20 minutes. Samples were imaged with Zeiss Axio Observer.Z1 inverted microscope (Zeiss LSM800, Oberkochen, Germany) using Plan-Apochromat 63x/1.40, WD 0.19 mm oil immersion objective. We used fibronectin antibody to visualize the patterned shapes for better tracking the single cells. A phalloidin antibody was used to visualize the actin (Table S1).

Antibodies

Table S1: Antibodies used in immunostaining. Antibodies used in immunostaining were diluted in 1.5% BSA, 0.1% Triton-X /PBS buffer.

Antibody	Manufacturer	Method	Dilution used
anti-vinculin	Merck, clone hVIN, V9131, RRID:AB_477629	Immunostaining	1:100
anti-FAK-pY397	Abcam, ab81298 [EP2160Y], RRID:AB_1640500	Immunostaining	1:100
anti-paxillin	BD Biosciences, 349/Paxillin, 610051, RRID:AB_397463	Immunostaining	1:100
anti-paxillin pY31	Thermo Scientific, 44-720G	Immunostaining	1:100
GFP antibody	Sicgen AB0020-200	Western blot	1:1000
Integrin β1 (D6S1W)	Cell signaling technology, #34971	Immunostaining	1:100
CD29 clone 9EG7	BD Pharmingen, Lot# 8309642	Immunostaining	1:100
Anti-fibronectin	Sigma, Lot# 098M4855V		
Actin	Millipore, MAB 1501R, RRID: AB_2223041	Western blot	1:2000
Alexa Fluor 568 phalloidin	Life Technologies	Immunostaining	1:40
Alexa Fluor 568 goat anti–rabbit IgG	Life Technologies A11011	Immunostaining	1:200
Alexa Fluor 568 goat anti–mouse IgG	Molecular probes, A11004	Immunostaining	1:200
Alexa Fluor 594 goat anti–rat	Life Technologies	Immunostaining	1:200

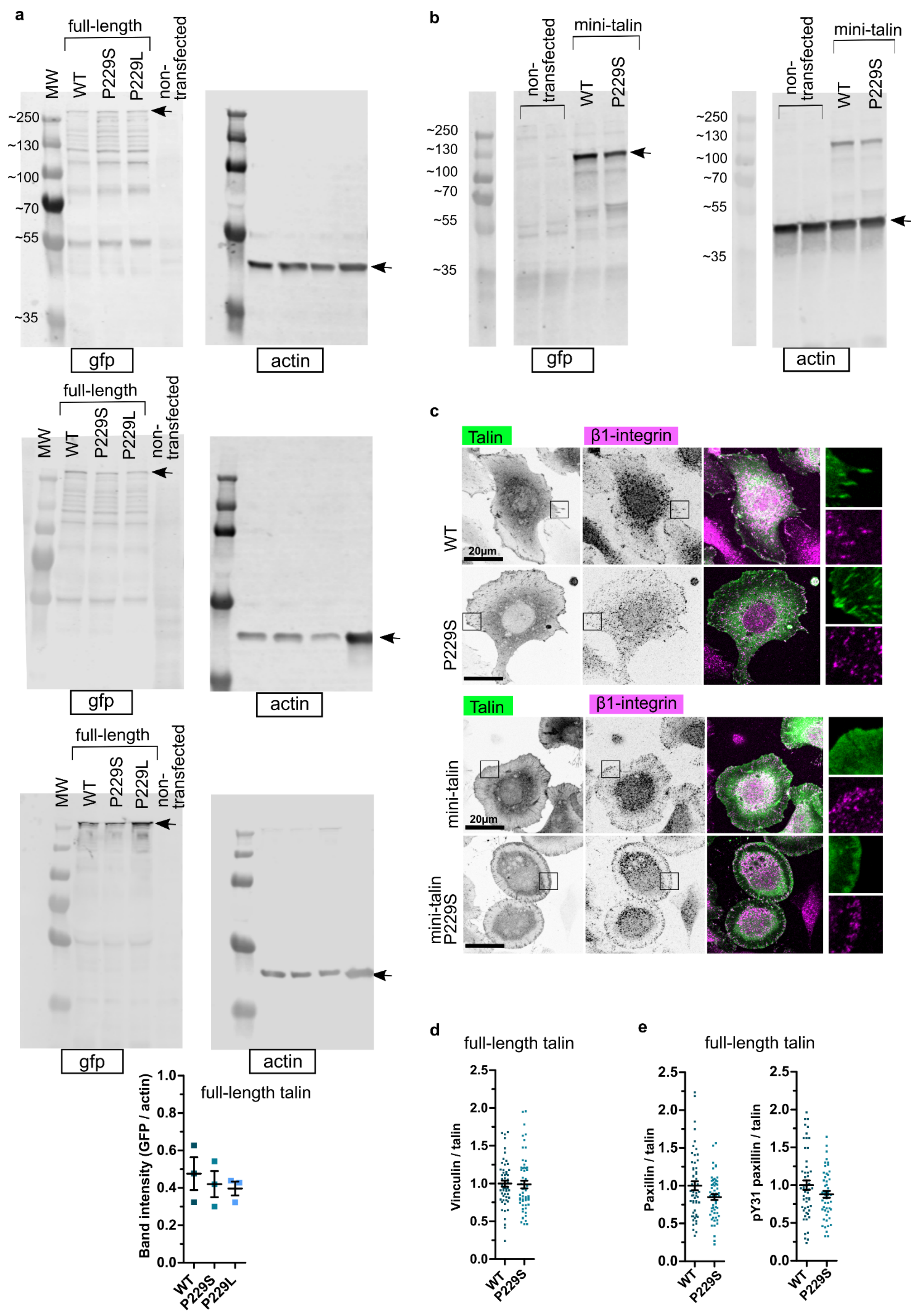

Figure S1. Talin variants are correctly expressed but differ from WT in their colocalization with paxillin. a,b) Western blot analysis and quantification for the different talin forms (full-length and mini-talin)

to confirm the correct expression level and size. Blot are presented from three separate experiments. For each, the same blot is stained by actin ~40 kDa (right). **c)** Representative images of cells expressing full-length/truncated talin proteins. Total β1-integrin is shown in the staining. **d,e)** vinculin/talin (d) paxillin/talin, pY31-paxillin/talin (e) intensity ratios for the cells transfected with full-length talin WT and P229S. n~ 50 cells pooled from three independent experiments. The data in d) and e) are normalized to the WT in each set. The statistical analysis was done by t-test Mann-Whitney test; *P<0.05, **P<0.01, ***P<0.001. Data represent the mean values with SEM.

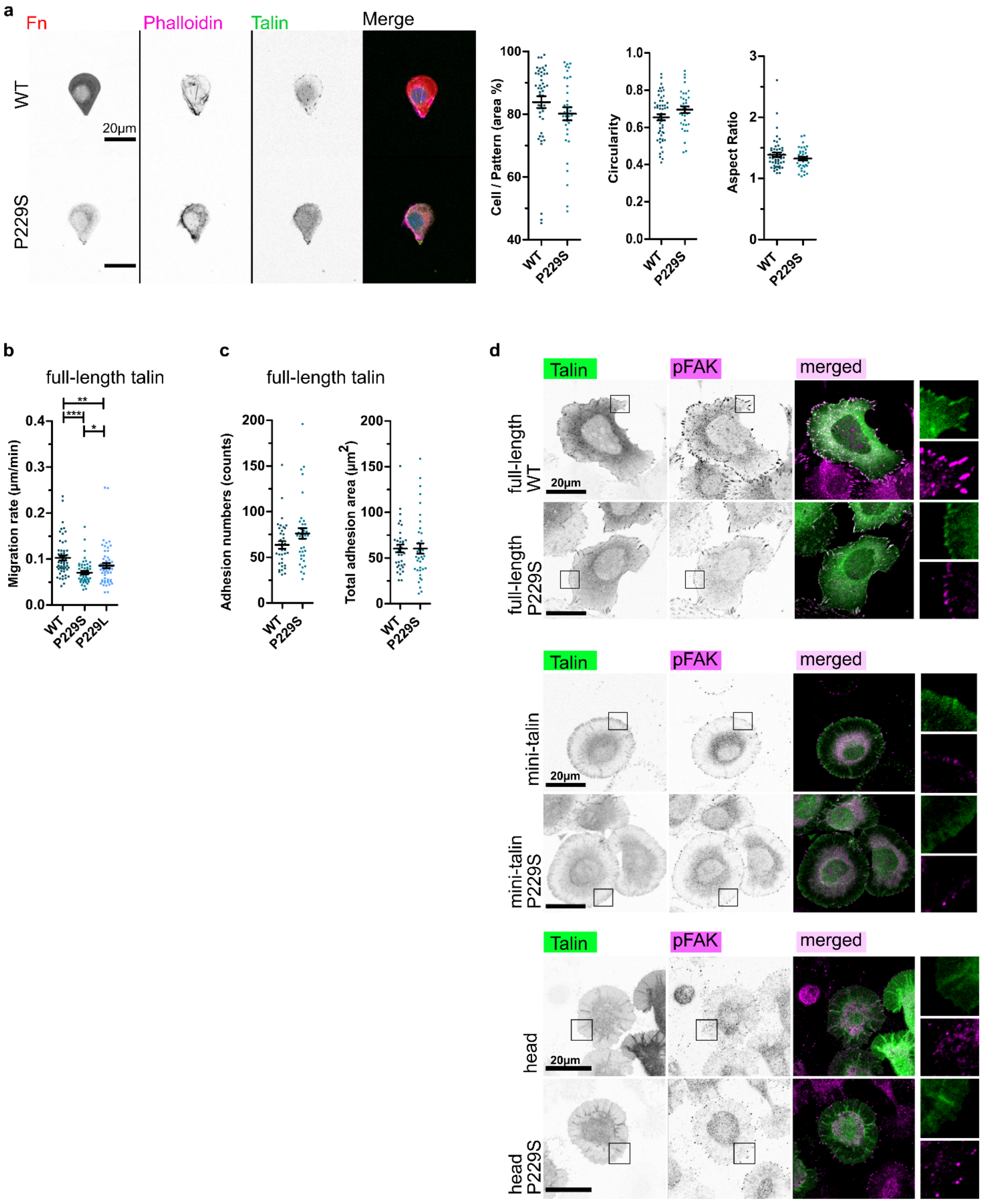

Figure S2: Single cell transfected with WT and/or P229S cultured on ice-cream shaped patterned areas of different sizes. a) The representative confocal image stained with fibronectin (Fn, red, patterned area), phalloidin (magenta), talin (green) and DAPI (blue) (left). Cell area (%), circularity and aspect ratio (right) of the single cells analysed from the 400 μm^2 patterned area. n~25 cells pooled from four independent experiments. **b)** Random migration analysis of the cells transfected with full-length talin WT, P229S and P229L from surface coated with vitronectin (10 µg/ml). n~60 cells pooled from three independent experiments. The experiments performed in 0% FBS medium. **c)** Adhesion number and total area quantification from the

sample immunostained against paxillin. **d)** Representative images of cells expressing full-length/truncated talin proteins. pFAK is shown in the staining. The statistical analysis in a), b) and c) was done by t-test Mann-Whitney test; *P<0.05, **P<0.01, ***P<0.001. Data represent the mean values with SEM.
